# Striving for better health through health research in post-conflict Timor-Leste

**DOI:** 10.1186/1478-4505-10-13

**Published:** 2012-04-10

**Authors:** Nelson Martins, Zoe Hawkins

**Affiliations:** 1 Minister of Health, Democratic Republic of Timor-Leste, Ministry of Health, Caicoli, Dili, Timor-Leste; 2 Former advisor to the Ministry of Health, Timor-Leste for mental health and health research, General Hospital Mental Health Department, Austin Hospital, 145 Studley Road, Heidelberg VIC 3084, Australia

**Keywords:** Health research, Timor-Leste, Research capacity, Developing country

## Abstract

The Cabinet of Health Research and Development (CHRD) has recently been established as the first health research institute in one of the world's newest nations, Timor-Leste. We discuss the development of this initiative to build health research capacity within the context of Timor-Leste's health system, history and future goals.

## Background

Strengthening research capacity in developing countries is "one of the most powerful, cost-effective, and sustainable means of advancing health and development", according to the 1990 report of the International Commission on Health Research for Development [1]. The capacity to conduct high-quality and relevant health research and use the results to implement evidence-based policies clearly benefits health systems and advances health in general. This is true for any country, rich or poor. But the challenges to building this research capacity are far greater for developing countries, which struggle with low resources, poor infrastructure and diminished capacity.

In the two decades following this report the world has seen huge growth in aid initiatives aimed at strengthening health-systems in low and middle income countries [2] and has heard increasing calls to strengthen their health research capacities [3-5]. Global partnerships for health research have been forged [5] and some of the relevant countries continue to report and reflect on endeavors to improve their own health research capacities [6-8]. There remains, however, a paucity of published work regarding concepts for building research capacity in developing countries. This paper is therefore novel in describing such an initiative and may serve to stimulate similar papers and debate from other nations.

Developing countries carry 90% of the global burden of disease yet receive less than 10% of the global spending on health research [9]. A minimal amount of primary research is carried out in developing countries and this may be biased against for publication [10] or simply not published at all. Current systematic reviews do not generally reflect developing world priorities, and findings from systematic reviews provided by the developed world may not be feasible to implement in countries with fewer resources [11]. Thus developing countries simply cannot rely on the research findings of the developed world and must act locally to build their own research capacity.

## Main text

### Timor-Leste

Situated on the eastern half of the island of Timor, between Australia and Indonesia, Timor-Leste has been independent since May 2002 following a 24-year occupation by neighbouring Indonesia. The Ministry of Health is based in the capital, Dili, and services a population of one million.

One of the world's newest nations following its independence, Timor-Leste continues to struggle against extreme poverty, high maternal and child mortality rates, malaria, tuberculosis and malnutrition [12]. Much of the population resides in rural and inaccessible mountainous areas. Access to clean water and knowledge of hygiene and sanitation is poor in many rural areas. As is typical of such countries there is more to do and fewer resources with which to do it. Factors necessary to increase research capacity are many, but in low-resource developing countries, it is political will that takes primacy [3]. The commitment of the government becomes the central and almost deciding factor in the success of efforts to strengthen research capacity.

### Striving for better health through health research

The Cabinet of Health Research and Development was conceived by the Minister of Health as an essential component for developing the health system of the country, and fostering an environment in which good-quality and ethical health research could flourish. The vision of the CHRD is 'striving for better health through health research', and through this is sought a health system where evidence-based practice is the norm, not only in direct medical interventions but within policy and programs generally [13].

Prior to the establishment of the CHRD, a small number of health research projects were carried out in Timor-Leste, which were exclusively foreign-led and often conducted by postgraduate students from external universities. A notable exception was the regular in-country research conducted by the non-governmental organisation Health Alliance International (HAI), which was used to evaluate the effectiveness of their maternal and child health interventions [14]. Early health research in Timor-Leste did not focus on sustainability or capacity-building of local staff, and processes to retain and utilize results within Timor-Leste were lacking. As a result, much valuable data was lost when researchers returned to their own countries, and thus could not be used to guide policy and practice, or to inform the Ministry of Health or health-related NGO programs in any way.

In addition there were no procedures for obtaining ethics approval for projects either initiated from within Timor-Leste or from external institutions. While the Ministry of Health ensured that ethics approval had been granted by the relevant Institutional Review Board in a researcher's home country, this may not have taken into consideration the particular cultural or religious context or other specific ethical factors relating to potential subjects in Timor-Leste.

In creating the CHRD, a formal process for organizing, facilitating and promoting health research was established. This would include the development of a database of the countries' health research; a resource library with access to international journals; training and workshops for local staff; an ethics review board; and development of institutional partnerships between Timor-Leste and developed countries. The CHRD was also intended to conduct its own health research following identification of research priorities within the country.

Through these activities it was felt that long-term improvements in the health of the nation would arise. Robust and accurate data from rigorous research would be used to monitor and evaluate the impact and effectiveness of health interventions. The results would be used to develop more evidence-based policies and guidelines, resulting in a more responsive health system with efficient and effective service delivery.

### Early processes

A small team of two Timorese Ministry of Health staff and two International Advisors began the planning process, and faced immediate and significant practical challenges. No funding was available and it was not possible to secure office space or equipment within the already overcrowded and under-resourced Ministry of Health. Meetings with the Minister took place in various appropriated offices of other departments. There was no legal basis for the team's work. Progress was further slowed by other work deadlines, regular extended power-cuts and flooding of the premises.

In September 2009 at the World Health Organisation (WHO)'s 62^nd ^session of the Regional Committee for South East Asia, a commitment was made by WHO to assist with the development of health research systems in specific countries of the South East Asia region, including Timor-Leste. As part of this commitment a senior researcher from Indonesia was contracted by WHO to assist the establishment of the CHRD through a series of three-month placements.

**Figure 1:**
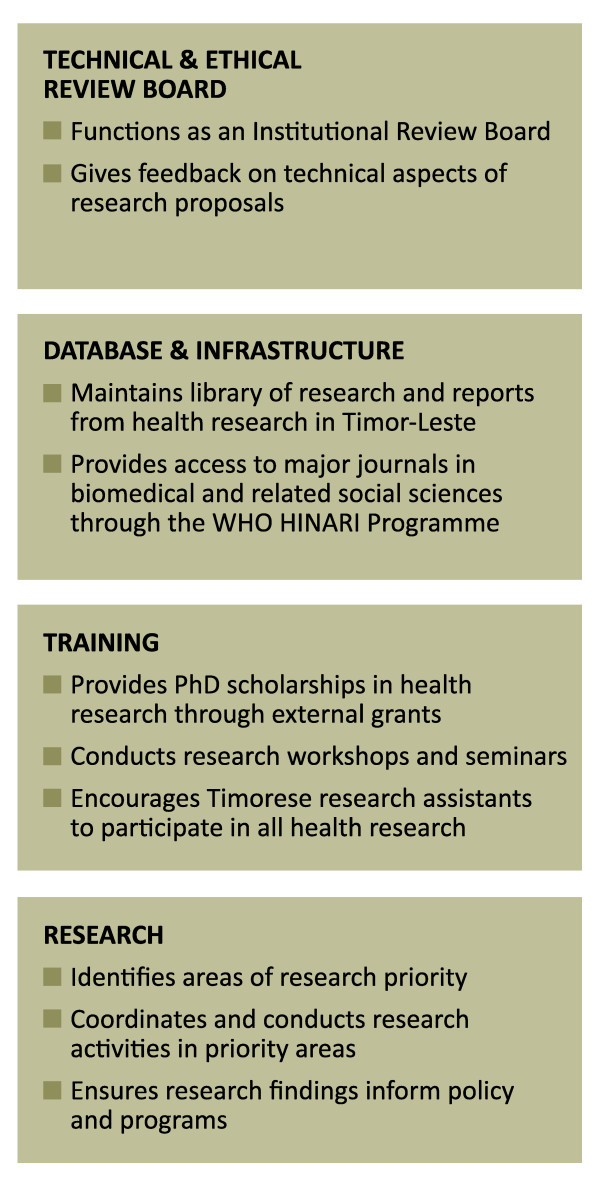
**Objectives and Functions of the CHRD**.

Further preliminary activities involved sourcing further funding, developing the structure of the organisation and drafting early policy. The legal basis for the Cabinet was provided through Ministerial decree rather than through the more lengthy process of amending the Organic Law. The CHRD currently sits within the overall structure of the Ministry of Health, but will eventually stand as an independent research institute. The objectives and functions of the CHRD are summarised in Figure 1.

### Funding

Through the new Health System Strengthening Project funded jointly by AusAID through the World Bank and the European Union, a medium term budget has been allocated to the CHRD. The 1990 report of the Commission on Health Research for Development [1] proposed that low and middle income countries should allocate 2% of national health expenditures and 5% of externally-funded programmes to research and capacity strengthening. This requires the explicit support of the government, as well as strong and open relationships with external aid programmes for negotiations around budgeting and resource allocation.

### Developing a research partnership

Strengthening research capacity in developing countries requires international and national cooperation [15]. This approach to capacity building requires a larger financial investment than both on-the-job experience and the formalised post-graduate training of individuals. However the likelihood of sustainability is greater with this approach and the research focus progresses from basic research skills through to an emphasis on program and policy [16].

The Menzies School of Health Research and the CHRD have collaborated in a twinning arrangement through which the CHRD will be supported in its health research system development. This arrangement incorporates the four broad principles of a truly co-operative research partnership proposed by Costello and Zumla [17], including mutual trust and shared decision making; national ownership; emphasis on translating research findings into policy and practice; and development of national research capacity.

Other national links have also been made with the Faculty of Medicine and Health Science at the University of Timor-Leste, and the Faculty of Public Health at UNPAZ (*Universidade da Paz*).

## Discussion

Through the establishment of the CHRD's Technical and Ethical Review Board in 2010, a formal, standardised process has been developed to ensure that the rights and welfare of subjects participating in health research are fully protected. Board members also provide feedback on technical aspects of the proposals.

The CHRD underlines the importance of including Timorese nationals as research assistants, even in studies originating outside of the country. This strengthens local capacity, and enthuses national staff to conduct research projects of their own. This 'learning by doing' approach is considered by Lansang and Dennis [16] to be an effective method to complement the approach of formal graduate or post-graduate training. In Timor-Leste, formal graduate training in health research was initiated through PhD scholarships offered to Timorese nationals by the Menzies School of Health Research in Darwin, Australia. Together, the 'learning by doing' and formal academic training approaches provide the backbone of human resource development for national research systems [16].

Major hurdles have been overcome to successfully establish Timor-Leste's first health research institution. Ongoing development and sustainability of the CHRD will see many further challenges. Consistent and long-term funding of research initiatives from the government and externally funded aid programmes is essential. Given the pressing need for service delivery as well as budget limitations, this is by no means easy to negotiate. The importance of financing health research can be difficult to argue when urgent health services are required and external project funds are time-limited. Further, the Ministry of Health must provide evidence of improvements in health indicators during their period of office, whilst the beneficial effects of health research may not be seen for many years. Internal debates on this issue exist within government with some people of the opinion that limited resources should be allocated to service provision rather than research. Also, changes of government may abruptly change priorities, policies and practices, which could disrupt the continuity of health research system development. Clearly, a committed, long-sighted vision of government and external aid agencies is essential and this requires ongoing education, careful negotiations and firm, unified political backing.

## Conclusions

In its first three years the Cabinet of Health Research and Development in Timor-Leste has grown from a concept into a functioning institution with its own legal framework, policy, staff, offices, and budget. Building long-term research partnerships is critical, as is the incorporation of CHRD activities into the Ministry of Health 30-year strategic plan. Research must be promoted as a viable career option, which may require schemes such as research awards and salary supplements [16]. Resource flows must allocate greater percentages of the health budget into health research, which requires determined and consistent political motivation. As a new initiative for Timor-Leste, the CHRD will continue on the strong foundations that have been laid to strengthen and grow, fulfil its objectives and ultimately improve the health of its people.

## Abbreviations

CHRD: Cabinet of Health Research and Development; WHO: World Health Organisation

## Competing interests

Dr Nelson Martins is the Minister of Health for Timor-Leste.

## Authors' contributions

NM initiated the CHRD project and invited ZH to document its development and progress. Both authors jointly decided on the scope and outline of the paper. ZH prepared the outline of the paper and worked jointly with NM on its revisions. ZH is the corresponding author. All authors read and approved the final manuscript.

## Authors' information

NM is the current Minister of Health for the Democratic Republic of Timor-Leste.

ZH worked for two years (2009-10) as Advisor to the Ministry of Health, Timor-Leste for mental health and health research. She is currently working as a Psychiatry Registrar at the Austin Hospital in Melbourne.
